# Increasing plasma L-kynurenine impairs mitochondrial oxidative phosphorylation prior to the development of atrophy in murine skeletal muscle: A pilot study

**DOI:** 10.3389/fphys.2022.992413

**Published:** 2022-09-30

**Authors:** Victoria R. Palzkill, Trace Thome, Ania L. Murillo, Ram B. Khattri, Terence E. Ryan

**Affiliations:** ^1^ Department of Applied Physiology and Kinesiology, Gainesville, FL, United States; ^2^ Center for Exercise Science, Gainesville, FL, United States; ^3^ Myology Institute, University of Florida, Gainesville, FL, United States

**Keywords:** physical function, weakness, mitochondria, energetics, metabolism

## Abstract

**Introduction:** L-Kynurenine (L-Kyn), a product of tryptophan (Trp) catabolism, has been linked with impairments in walking speed, muscle strength/size, and physical function. The purpose of this pilot study was to develop a dietary model that elevates plasma L-Kyn levels in mice and characterize its impact on muscle health and function.

**Methods:** Four-month-old C57BL6J male mice were randomized to either a L-Kyn supplemented (150 mg/kg) or chow diet for 10 weeks. Plasma L-Kyn and Trp levels were measured via mass spectrometry. Primary outcomes included assessments of muscle weights, myofiber cross-sectional area (CSA), nerve-stimulated contractile performance, and mitochondrial oxidative phosphorylation (OXPHOS) and hydrogen peroxide (H_2_O_2_) production. Additional experiments in cultured myotubes explored the impact of enhancing L-Kyn metabolism.

**Results:** Mice randomized to the L-Kyn diet displayed significant increases in plasma L-Kyn levels (*p* = 0.0028) and the L-Kyn/Trp ratio (*p* = 0.011) when compared to chow fed mice. Food intake and body weights were not different between groups. There were no detectable differences in muscle weights, myofiber CSA, or contractile performance. L-Kyn fed mice displayed reductions in mitochondrial OXPHOS (*p* = 0.05) and maximal ADP-stimulated respiration (*p* = 0.0498). In cultured myotubes, overexpression of peroxisome proliferator-activated receptor-gamma coactivator 1 alpha prevented atrophy and proteolysis, as well as deficits in mitochondrial respiration with L-Kyn treatment.

**Conclusion:** Dietary feeding of L-Kyn increases plasma L-Kyn levels and the L-Kyn/Trp ratio in healthy male mice. Mitochondrial impairments in muscle were observed in mice with elevated L-Kyn without changes in muscle size or function. Enhancing L-Kyn metabolism can protect against these effects in culture myotubes.

## Introduction

L-Kynurenine (L-Kyn) is a metabolite derived from the degradation of the essential amino acid tryptophan (Trp). The kynurenine pathway begins with the conversion of Trp into N-formylkynurenine by the enzymes indoleamine 2,3 dioxygenase or tryptophan 2,3-dioxygenase (TDO), followed by the removal of formate to produce L-Kyn. Metabolism of L-Kyn involves either the biotransformation of L-Kyn to kynurenic acid (KA) by kynurenine aminotransferases (KATs) or the *de novo* synthesis of nicotinamide adenine dinucleotide (NAD^+^), a critical electron carrier required for effective energy transduction (Castro-Portuguez and Sutphin, 2020).

Several recent human studies have reported strong associations between L-Kyn levels and frailty, muscle atrophy, and neuromuscular junction degeneration (Marcos-Perez et al., 2017; Rattray et al., 2019; Jang et al., 2020; Westbrook et al., 2020). Jang et al. (2020) reported high serum kynurenine levels in frail older adults, even when adjusted for BMI, sex, and age. In this study, regression analysis identified that L-Kyn levels were strongly associated with a frailty index and physical function measures including grip strength, gait speed, and chair stand. Consistent with these results, Westbrook et al. (2020), using a larger sample size, reported that both non-frail and frail older adults had higher levels of L-Kyn and several downstream metabolites when compared to young adults. Logistic regression analysis performed in the Westbrook *et al.* study confirmed a significant relationship between frailty and L-Kyn/tryptophan ratio. Despite compelling associations between elevated kynurenines and muscle health/function, a cause-effect relationship has not been established.

One previous study reported that increasing L-Kyn levels in mice with intraperitoneal injections causes muscle atrophy in young mice (Kaiser et al., 2019a). In cultured muscle cells, L-Kyn treatment has been reported to increase mitochondrial reactive oxygen species (ROS) and impair mitochondrial respiration (Kaiser et al., 2019b; Thome et al., 2019). Furthermore, L-Kyn treatment in a culture model has been shown to cause motor neuron death via ATP depletion (Westbrook et al., 2020). In *drosophila*, lowering L-Kyn levels by inhibition of TDO2 improved motor function in neurodegenerative conditions (Breda et al., 2016). These findings are interesting considering that recent work has shown that elevated denervation (loss of myofiber innervation by motor neurons) and the associated mitochondrial dysfunction in muscle are pivotal drivers of muscle atrophy and weakness (Piasecki et al., 2018; Sonjak et al., 2019a; Sonjak et al., 2019b). Thus, it is plausible that lowering L-Kyn levels could ameliorate muscle impairment in conditions with elevated kynurenines. As recently reviewed by Martin et al. (2020), skeletal muscle is also a large organ responsible for converting L-Kyn to KA and the enzymes responsible are altered by muscle activity/inactivity.

In this pilot study, we sought to test the impact of a kynurenine-supplemented diet on skeletal muscle contractile function and size, and mitochondrial health. It was hypothesized that mice fed a kynurenine-supplemented diet would exhibit muscle atrophy, weakness, and deficits on mitochondrial respiratory function.

## Materials and methods

### Animal use and models

Ten 16-week-old C57BL/6J male mice were purchased from Jackson Laboratory and were housed with five mice/cage in a temperature (22°C) controlled room with 12-h light/dark cycles. Mice were randomly assigned to receive L-Kyn supplemented diet (150 mg/kg, added to Envigo Teklad Global 18% Protein Rodent Diet 2,918) or remained on normal chow (Envigo Teklad Global 18% Protein Rodent Diet 2,918 irradiated pellet), *n* = 5/group. Diets were provided ad libitum for 10 weeks. Body weight and food consumption was measured each week. All animal experiments adhered to the Guide for the Care and Use of Laboratory Animals from the Institute for Laboratory Animal Research, National Research Council, Washington, D.C., National Academy Press. All procedures were approved by the Institutional Animal Care and Use Committee of the University of Florida.

### Plasma metabolomics

Blood was collected in heparin-coated capillary tubes via a small ∼1 mm tail snap and then centrifuged at 4000rpm for 10 minutes at 4°C. The resulting plasma was collected and immediately snap frozen in liquid nitrogen then stored at −80°C until processing. Samples were transported on dry ice to the Southeast Center for Integrated Metabolomics at the University of Florida and processed for targeted metabolomics (LC-MS/MS) as previously described (Kim et al., 2021; Thome et al., 2021).

### Nerve-mediated muscle contractile function

Muscle function was assessed using nerve-mediated contraction of the extensor digitorum longus (EDL) muscle as previously described with slight modifications (Koh and Brooks, 2001). Mice were anaesthetized with an intraperitoneal injection of xylazine (10 mg/kg) and ketamine (100 mg/kg). The distal EDL tendon was carefully isolated and tied using a 4-0 silk suture and cut below the suture. The mouse was placed prone on a heated (37°C) platform and the patellar tendon was secured to an immobilized pin attached to the board. The suture attached to the distal EDL tendon was secured to a force-length transducer (Cambridge Technology; Model: 2,250). Two electrodes (Chalgren, Cat. No. 111-725-24TP) were placed on either side of the peroneal nerve and connected to a stimulator (701A stimulator; Aurora Scientific) with the voltage set to 15V. Data collection was performed using the DMC program (version v5.500, Aurora Scientific). Optimal length was determined by recording force produced by twitch contractions while incrementally increasing muscle length with 1 minute rest between twitches. Once optimal length was obtained, force frequency curves were generated by stimulation at 1, 25, 50, 75, 100, 125, 150, and 175 Hz (spaced 1 minute apart) using square wave pulses of 0.5 s. Specific force was calculated by normalizing absolute forces to muscle weight. Fatiguability was assessed through a series of 80 contractions at 50 Hz (using 0.5 s pulse) performed every 2 seconds.

### Tissue harvest

Immediately following muscle contraction measurements, muscle from both hind limbs were carefully dissected and weights obtained. Soleus and EDL from the left limb were placed in optimal cutting temperature (OCT) compound and frozen in liquid nitrogen-cooled isopentane. The gastrocnemius muscle was placed on ice in Buffer A (PBS supplemented with EDTA (10 mM), pH = 7.4) for mitochondrial isolation.

### Isolation of skeletal muscle mitochondria

Skeletal muscle mitochondria were isolated from the left gastrocnemius muscle as previously described (Thome et al., 2021). Protein concentration of the final mitochondrial resuspension was determined using bicinchoninic acid protein assay kit (ThermoFisher Scientific, Cat. No. A53225).

### High-resolution respirometry

High-resolution respirometry was performed using an Oroboros Oxygraph-2k (O2K) to measure oxygen consumption (*J* O _2_) at 37°C. Twenty micrograms of mitochondria were added to the O2K chamber in 2ml of buffer D (105 mM K-MES, 30 mM KCl, 1 mM EGTA, 10 mM K_2_HPO_4_, 5 mM MgCl_2_-6H_2_O, 2.5 mg/ml BSA, pH 7.2) supplemented with 5 mM creatine monohydrate. Mitochondria were energized by the addition of 5mM pyruvate, 2.5 mM malate, and 0.2 mM octanoylcarnitine. Next we added a clamp system containing ATP (5 mM), phosphocreatine (PCr) (1mM), and creatine kinase (CK) (20 U/ml) which couples the interconversion of ATP and ADP to that of phosphocreatine (PCr) and free creatinine, to titrate the extra mitochondrial ATP/ADP ratio, thus free energy of ATP hydrolysis (ΔG_ATP_), to measure mitochondrial oxygen consumption at physiologically relevant levels of energy demand as done previously (Fisher-Wellman et al., 2018). Exogenous cytochrome c (10 mM) was used to assess outer-membrane integrity of isolated mitochondria and samples with more than a 20% increase in oxygen consumption were excluded from this study. The ΔG_ATP_ was plotted against the corresponding *J* O _2_, and the slope was used to represent conductance throughout mitochondrial oxidative phosphorylation (OXPHOS), where lower conductance indicates impaired mitochondrial energetics.

### Mitochondrial H_2_O_2_ production and electron leak

Mitochondrial H_2_O_2_ production was measured fluorometrically using Amplex Ultra Red (AUR) and horseradish peroxidase (HRP) in Buffer D using identical substrate conditions described above in a Horiba Quantmaster 400 spectrofluorometer (Excitation/Emission = 530/590 nm) as described previously (Thome et al., 2021). All reactions were done at 37°C with a 250 µl reaction volume and 20 μg of mitochondrial protein. Fluorescence values were converted to pmoles of H_2_O_2_ using a standard curve and rates of H_2_O_2_ production were calculated as pmoles/min/mg. An estimation of electron leak was calculated by dividing *J*H_2_O_2_ by *J* O _2_ and expressed as a percentage.

### Immunofluorescence microscopy

Skeletal myofiber cross-sectional area (CSA) was assessed by immunofluorescence microscopy as previously described (Berru et al., 2019). Images were obtained at ×20 magnification using an Evos FL2 Auto microscope (ThermoFisher Scientific) and tiled/merged images of the entire muscle section were used for analysis. Quantification of myofiber CSA was performed using MuscleJ (7), an automated analysis software developed in Fiji.

### Muscle cell culture

Murine C2C12 myoblasts were obtained from ATCC (Cat. No. CRL-1772). Myoblasts were grown in Dulbecco’s modified Eagle’s medium (DMEM) + GlutaMAX (GIBCO, Cat. No. 10569) supplemented with 10% fetal bovine serum (FBS; VWR cat. no. 97068), and 1% penicillin/streptomycin (GIBCO, Cat. No. 15140) in standard culture conditions (37°C in 5% CO_2_). Once cells reached ∼90% confluence, myotube differentiation was initiated via serum withdrawal by placing cells in differentiation medium [DMEM supplemented with 2% horse serum (GIBCO, Cat. No. 26050), 1% penicillin-streptomycin, and 1% insulin-transferrin-selenium (GIBCO, Cat. No. 41400)]. Differentiation medium was changed every 24 h. Myotubes were treated on day five of differentiation with DMSO (vehicle control) or 15μM L-Kynurenine (equal volume) for 18-h in differentiation medium. For experiments involving adenovirus infection for genetic manipulation, purified viruses containing either GFP or Peroxisome proliferator-activated receptor-gamma coactivator 1 alpha (PGC1α) were purchased from Vector Biolabs (Malvern, PA). Myotubes were infected with adenovirus on day three of differentiation using a multiplicity of infection (MOI) of 100. Adenovirus infection was confirmed by visual inspection of GFP and quantitative PCR.

### RNA isolation and quantitative real-time polymerase chain reaction

Total RNA was extracted from myotubes 48-h after adenovirus infection using TRIzol (Invitrogen, Cat. No. 15-596-018) for lysis and Direct-zol RNA MiniPrep kit (Zymo Research, Cat. No. R2052) following the manufacturer’s instruction. cDNA was generated from 500 ng of RNA using the LunaScript RT Supermix kit (New England Biolabs, E3010L) according to the manufacturer’s directions. Real-time PCR (RT-PCR) was performed on a Quantstudio 3 (ThermoFisher Scientific) using Luna universal qPCR master mix (New England Biolabs, M3003X) and the following primers: PGC1α (For myotube experiment: Forward-GAGTCTGTATGGAGTGACATCG, Reverse-TGTCTGTATCCAAGTCGTTCAC; For mouse experiment: Forward- AGA​AGT​CCC​ATA​CAC​AAC​CG, Reverse- TCG​CTC​AAT​AGT​CTT​GTT​CTC​AA), CCBL1/KAT1 (Forward-TCATGCTCAACCAGTACACC, Reverse-GTCACCAGCACATTCTTGAGT), CCBL2/KAT3 (Forward-CACGACACTCTGTGCATCAG, Reverse-GTCTTGCCAGCACTTCCTAT), GOT2/KAT4 (Forward-GTATTCCAACCCACCTCTCAA, Reverse-GCCATGCCTTTCACCTCTT) and L32 (Forward-TTCCTGGTCCACAATGTCAA, Reverse-GGCTTTTCGGTTCTTAGAGGA) was used as the housekeeping control. Relative gene expression was calculated using 2^−ΔΔCT^ from the Ad-GFP group.

### Myotube respiration

Following an 18-h treatment with 15μM L-Kyn or DMSO (equal volume), myotubes were rinsed with PBS, detached from flasks with 0.25% trypsin-EDTA (GIBCO Cat. No. 25200) and centrifuged at 800xG for 5 minutes to pellet myotubes. Myotubes were resuspended in 2.5 ml of buffer D supplemented with glucose (10 mM) and pyruvate (5 mM). Cells were loaded into the Oxygraph O2K chamber and respiration was measured at 37°C. Digitonin (10 μg/ml) was added to the chamber to permeabilize cells. Basal oxygen consumption (*J* O _2_) was measured followed by the addition of substrates malate (2.5 mM) and pyruvate (5 mM) to energize the mitochondria. Next, ADP (4 mM) was added to the chamber. Exogenous cytochrome C (10 mM) was added to confirm the integrity of mitochondrial membranes. Rates of oxygen consumption were normalized to protein content measured by BCA assay (ThermoFisher Scientific, Cat. No. A53225) and expressed as pmoles/sec/mg.

### Myotube area

Myotubes were treated with either 15μM L-Kyn or DMSO (equal volumes) in fresh differentiation medium for 18-h in standard culture conditions. Following treatment, myotube area was measured by quantifying myosin positive area as previously described (Berru et al., 2019). Cells were imaged using a ×20 objective and automated capture routines to image the inner 80% of the well on an Evos FL2 Auto microscope (ThermoFisher Scientific). Myosin-positive area was analyzed using custom written routines in Cell Profiler (Broad Institute, United States). All processing procedures were performed uniformly over the entire set of images using batch processing modes to remove any human bias.

### Myotube protein degradation

The rate of protein degradation was measured by quantifying the release of free tyrosine from incubated myotubes in the presence of cycloheximide (to inhibit protein synthesis) (Price et al., 1996; Enoki et al., 2017). Myotubes were incubated in Kreb’s buffer consisting of a mixture of mono (NaHCO_3_, 24 mM) and di-basic (NaH_2_PO_4_, 10 mM) sodium phosphate, NaCl (137 mM), and KCl (5 mM) along with glucose (10 mM) and cycloheximide (0.5 mM) at pH 7.4 and the buffer was gassed with 95% O_2_ and 5% CO_2_ at 37°C for 30 min. The buffer was then aspirated and replaced with fresh Kreb’s buffer and continuously incubated for 2.5 h maintaining 95% O_2_ and 5% CO_2_ at 37°C. Next, the Kreb’s buffer was collected and lyophilized overnight (Labconco, Cat. No. 700201000). Following lyophilization, 1 ml of acetonitrile:isopropanol:water (3:3:2 vol:vol:vol) was added to the sample and vortexed. The mixture was transferred into a 1.5 ml vial and centrifuged (at 13.2K rpm) at 4°C for 30 min. The supernatant was transferred into a new tube and lyophilized until dried. The dried lyophilized sample was then dissolved in 1 ml of acetonitrile:water (1:1 vol:vol) mixture, and vortexed. The mixture was centrifuged (at 13.2K rpm, 4°C) for 30 min and the supernatant was collected. The lyophilization process was repeated and the dry lyophilized powder was suspended in 50 μl nuclear magnetic resonance (NMR) solvent consisting of 50 mM phosphate buffer (pH 7.2) along with 0.5mM D6-4,4-dimethyl-4-silapentane-1-sulfonic acid (an internal standard), 2 mM EDTA, and 0.2% (w/v) sodium azide (Chenomx, Inc.) dissolved in deuterium oxide. All NMR experiments were carried out in the University of Florida, utilizing 600 MHz Bruker Avance Neo (Console II) NMR system (Bruker Biospin) equipped with a 1.7 mm CP TXI CryoProbe. To acquire one-dimensional proton (1 H) NMR spectra, the first slice of a NOESY pulse sequence (tnnoesy) (Ravanbakhsh et al., 2015) was utilized and all the experiments were performed at 25°C. The parameters utilized in this study to acquire and process 1D NOESY NMR spectra have been reported previously (Lohr et al., 2019; Osis et al., 2019). Chenomx NMR Suite 8.2 (Chenomx, Inc.) software was used to quantify tyrosine concentration. Rates of protein degradation (tyrosine release) were normalized to cellular protein content measured by BCA assay (ThermoFisher Scientific, Cat. No. A53225) and reported as nmols/hr/mg.

### KAT4/GOT2 enzyme activity assay

Mitochondrial-enriched lysates were prepared from C2C12 myotubes by homogenizing cells with a glass/Teflon homogenizer (Wheaton) followed by centrifugation at 800xG for 10 minutes. The resulting supernatant was transferred to a new tube and centrifuged again at 10,000xG to pellet the mitochondrial-enriched fraction. Mitochondrial-enriched pellets were gently re-suspended in mitochondrial isolation medium without BSA. The activity of kynurenine aminotransferase (GOT2/KAT4) was measured in assay buffer (20 mM HEPES, 100 mM KCl, 2.5mM KH_2_PO_4_, 2.5 mM MgCl_2_, 1% glycerol) containing 100 mM aspartate, 0.005 mM rotenone, 0.1 mM pyridoxal-5-phosphate, 2 U/ml malate dehydrogenase, and 0.2 mM NADH. Prior to initiating the reaction, mitochondria were lysed in CellLyticM (Millipore-Sigma, Cat. No. C2978) and 10 μg of the mitochondrial-enriched lysates were loaded in a 96-well plate containing 200 μl of the assay buffer. GOT2/KAT4 activity was determined by the rate of consumption of NADH (measured via auto-fluorescence at Excitation/Emission = 340/450 nm) after the addition of 12 mM alpha ketoglutarate. Fluorescence values were converted to pmoles of NADH *via* a standard curve.

### Statistical analysis

All data are presented as mean ± standard deviation (SD). Normality of data was tested with the Shapiro-Wilk test. Data were analyzed using two-tailed unpaired Student *t*-test or two-way ANOVA with Tukey’s post hoc testing when significant interactions were detected. All statistical analysis was performed in GraphPad Prism (version 9.2.0 (332), GraphPad Software, San Diego, CA, United States) with *p ≤* 0.05 being considered statistically significant.

## Results

### Consumption of an L-Kyn-supplemented diet increases plasma L-Kyn levels without altering food intake

**FIGURE 1:**
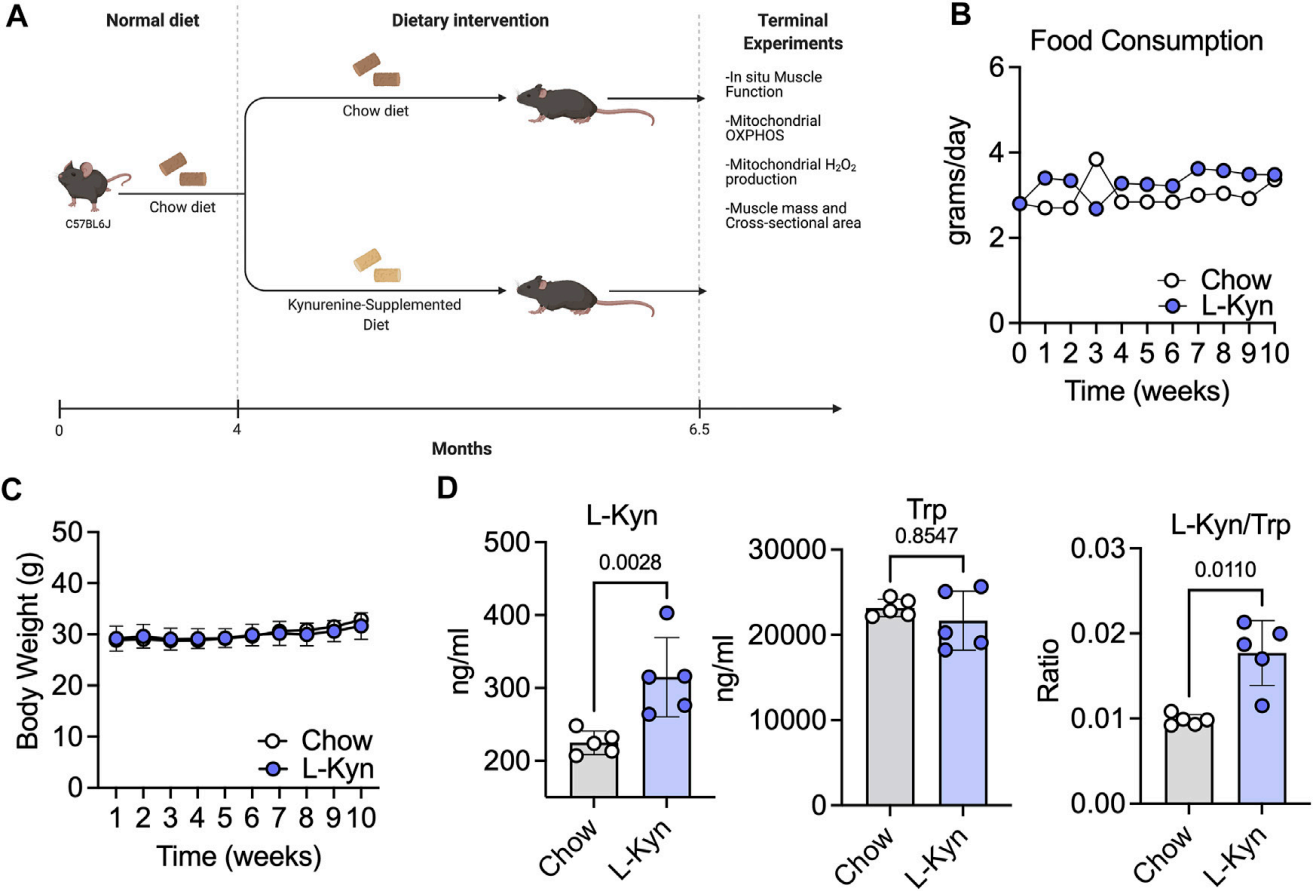
L-Kyn supplemented diet increases plasma L-Kyn levels and the L-Kyn/Trp ratio. **(A)**Graphical depiction of study design and outcome measures, created using Biorender.com. Weekly measures of **(B)** food consumption and **(C)** body weight over course of the intervention. **(D)** Plasma concentrations of L-Kyn, Trp, and the L-Kyn/Trp ratio assessed by liquid chromatography–mass spectrometry. Error bars represent SD. N = 5/group for all panels. Data in panels B and C were analyzed using a two-way ANOVA, whereas data in Panel D were analyzed using a two-tailed Student’s *t*-test.

To establish a model that increases plasma L-Kyn levels, we generated a L-Kyn-supplemented diet that was produced by adding 150mg of L-Kyn per kilogram of chow diet (generated by Envigo). Mice were provided either chow or L-Kyn-supplemented diet for 10 weeks (Figure 1A). Measurements of food consumption demonstrated that L-Kyn supplementation did not alter food intake (Figure 1B). Body weight was also unaffected by diet (Figure 1C). To test whether L-Kyn levels were altered by the diet, we performed targeted metabolomics using liquid chromatography–mass spectrometry (LC-MS) to measure L-Kyn and Trp levels in the plasma of mice. These analyses revealed that mice that consumed the L-Kyn-supplemented diet had significant increases in plasma L-Kyn (*p* = 0.0028) and the L-Kyn/Trp ratio (*p* = 0.011) compared to chow fed mice (Figure 1D). Importantly, the change in L-Kyn/Trp ratio was driven by the increase in L-Kyn as plasma Trp levels were not different between groups (*p* = 0.85).

### Ten weeks of L-Kyn-supplemented diet does not alter muscle size or contractile function

**FIGURE 2:**
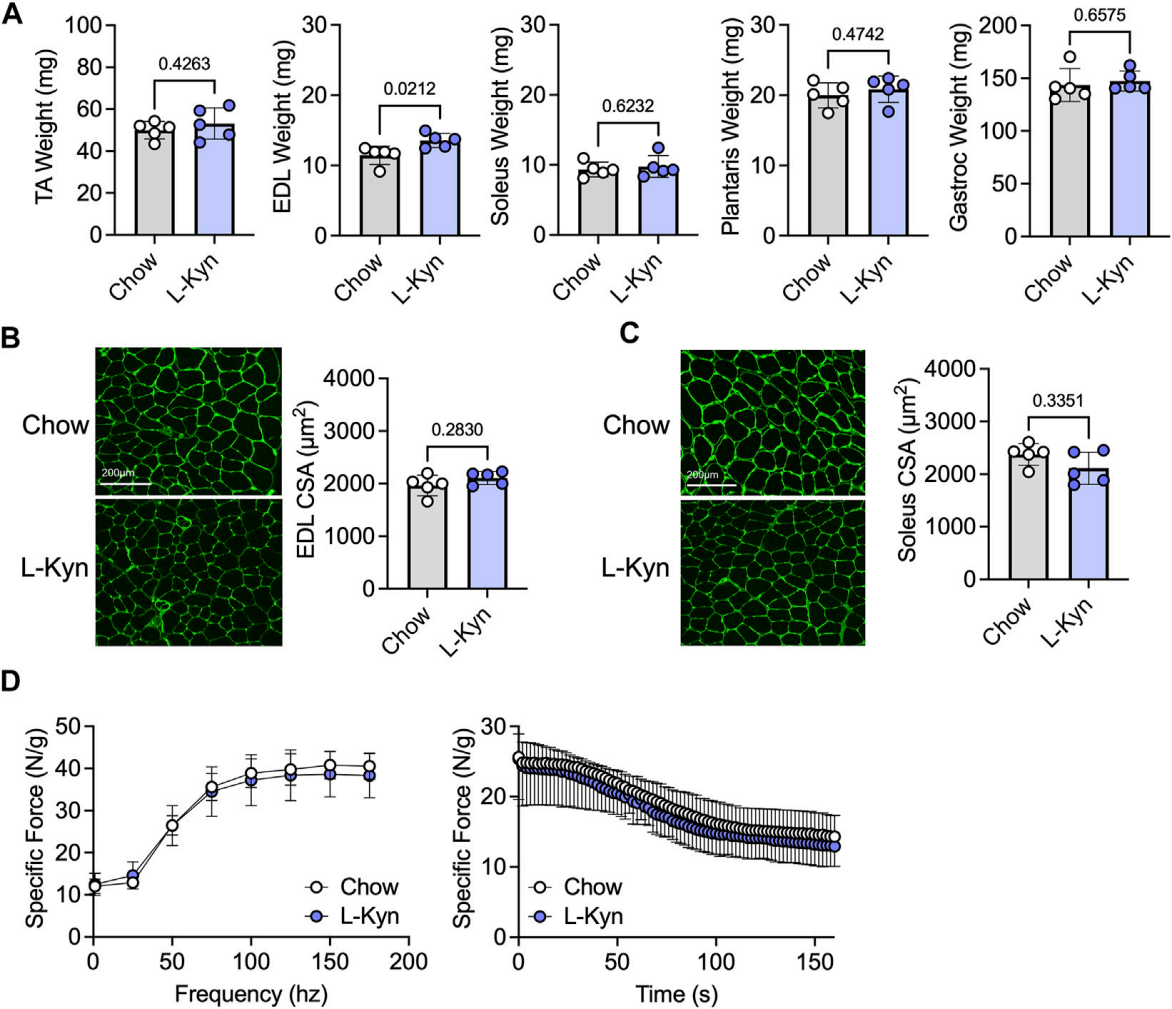
Dietary increases in L-Kyn does not alter muscle weight, cross-sectional area, or contractile function. **(A)** Quantification of muscle weights (mean of both limbs) following careful dissection. **(B)** Representative images and quantification of the mean myofiber cross-sectional area of the EDL muscle. **(C)** Representative images and quantification of the mean myofiber cross-sectional area of the soleus muscle. **(D)** Force frequency curves and muscle fatiguability measures generated by nerve stimulation. Error bars represent SD. N = 5/group for all panels. Data in panels A–C were analyzed using a two-tailed Student’s *t*-test. Data in panel D were analyzed with a two-way ANOVA.

Ten weeks of consumption of the L-Kyn-supplemented diet did not result in significant changes in muscle weight, measured across several hindlimb muscles, except for the EDL muscle which displayed a modest increase in weight in L-Kyn fed mice (Figure 2A). Similarly, the myofiber CSA in the predominantly fast fiber type EDL or slow fiber type soleus muscles was also unaffected by the diet (Figures 2B,C). Because previous work suggested that L-Kyn may have negative impact on the motor neuron (Westbrook et al., 2020), we also assessed muscle function using nerve-mediated stimulation. L-Kyn fed mice had no impairments in specific force or muscle fatiguability (Figure 2D).

### Mice fed a L-Kyn supplemented diet have impaired muscle mitochondrial OXPHOS

**FIGURE 3:**
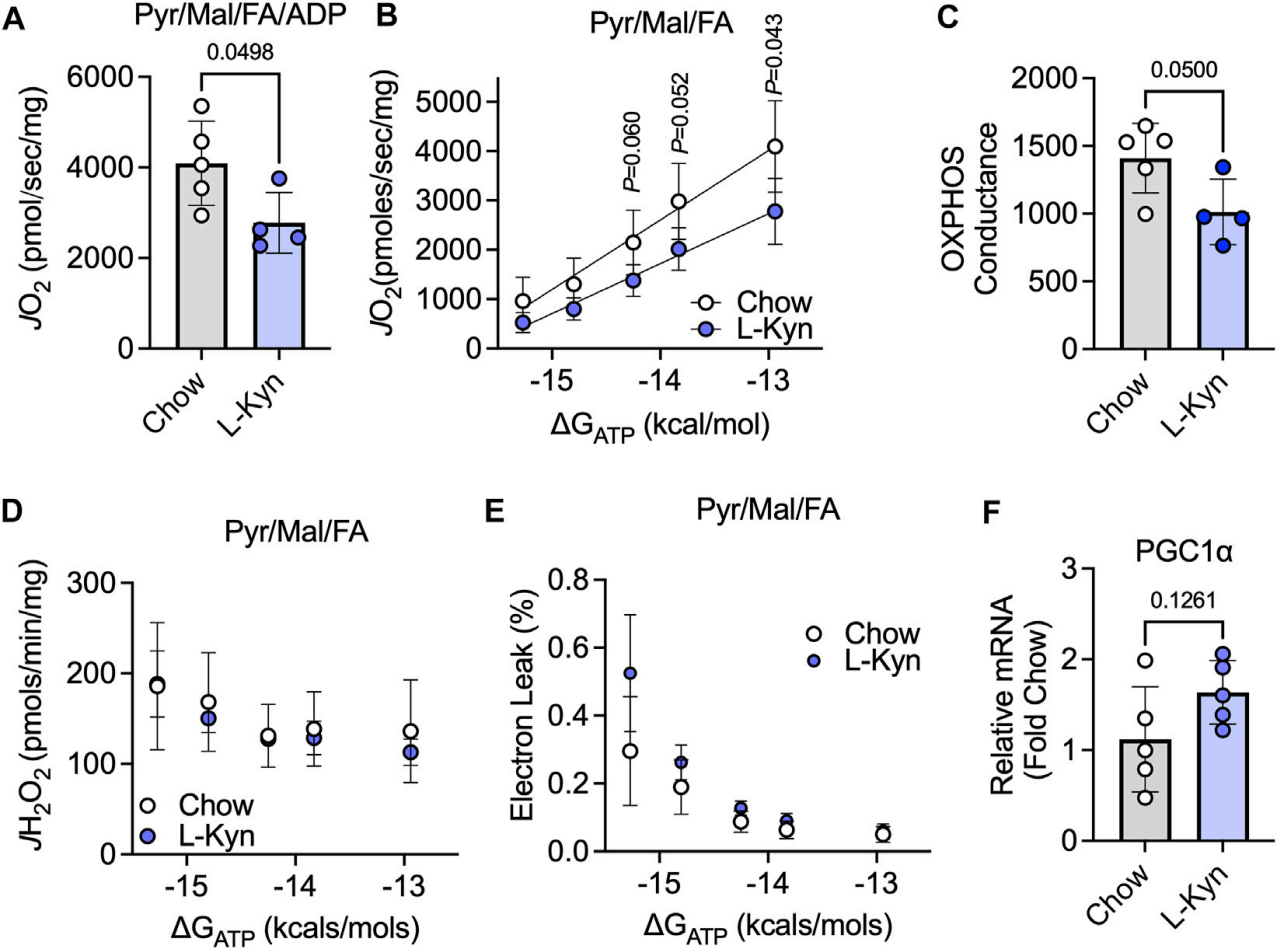
L-Kyn treatment impairs mitochondrial OXPHOS but does not affect H_2_O_2_ production in skeletal muscle. **(A)** Maximal ADP-stimulated respiration supported by pyruvate, malate, and octanoylcarnitine (Pyr/Ma/FA). **(B)** Oxygen consumption (*J* O _2_) as a function of the free energy for ATP hydrolysis (ΔG_ATP_) controlled by a creatine kinase clamp system. **(C)** Quantification of OXPHOS conductance (slope of relationship between *J* O _2_ and ΔG_ATP_). **(D)** Mitochondrial H_2_O_2_ production measured at each level of ΔG_ATP_ with Pyr/Mal/FA as substrates. **(E)** Estimated electron leak at each level of ΔG_ATP_ with Pyr/Mal/FA as substrates. **(F)** Relative mRNA levels of PGC1α in the gastrocnemius muscle. Error bars represent SD. N = 5/group for all panels. Data in panels A,C,D,E were analyzed using a two-tailed Student’s *t*-test. Data in panel B were analyzed with a two-way ANOVA.

To assess the impact of L-Kyn on skeletal muscle mitochondrial energetics, maximal ADP-stimulated respiration and a physiological assessment of the oxidative phosphorylation (OXPHOS) system were measured when fueling mitochondria with a combination of carbohydrates (pyruvate and malate) and fatty acids (octanoylcarnitine). Maximal mitochondrial respiratory function stimulated by addition of a 4mM ADP was decreased by ∼32% in L-Kyn fed mice compared to chow fed mice (*p* = 0.0498) (Figure 3A). Using a CK-clamp system to assess mitochondrial respiration under more physiologically relevant conditions, L-Kyn fed mice displayed lower levels of mitochondrial respiration at higher levels of energy demand (Figure 3B). The slope of the linear relationship between energy demand (ΔG_ATP_) and mitochondrial oxygen consumption (*J* O _2_), termed OXPHOS conductance, was significantly lower in L-Kyn fed mice (Figure 3C). The latter observation suggests that there is more resistance within the mitochondrial OXPHOS system, which in some cases, could manifest as increase electron leak and ROS production. However, this was not the case in the current study as no differences in mitochondrial H_2_O_2_ production (Figure 3D) or the estimated electron leak (Figure 3E) were detected. L-Kyn fed mice displayed a non-significant increase in PGC1α mRNA levels in the gastrocnemius muscle (Figure 3F), suggesting a possible compensatory response caused by increased L-Kyn levels.

### Expression of PGC1α protects against mitochondrial respiratory impairment and atrophy in cultured myotubes treated with L-Kyn

**FIGURE 4:**
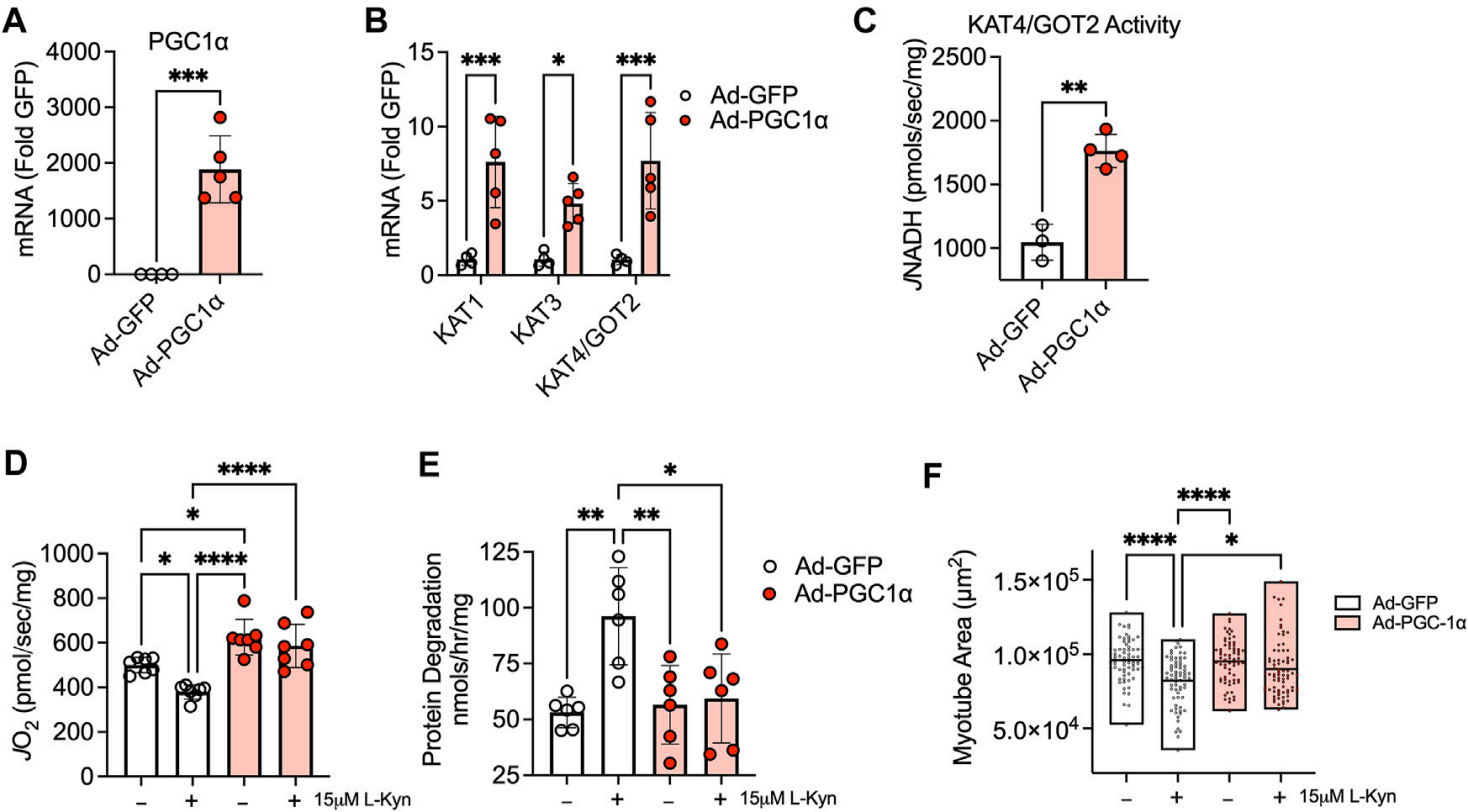
Adenovirus-mediated expression of PGC1α1 enhances L-Kyn metabolism and protects myotubes for atrophy and mitochondrial deficits. **(A)** Quantification of mRNA levels of PGC1α in myotubes infected with adenovirus encoding a GFP (control) or PGC1α. **(B)** Ad-PGC1α expression increased mRNA levels of cytosolic (KAT1 and KAT3) and mitochondrial (KAT4/GOT2) kynurenine aminotransferases. **(C)** Enzyme activity of KAT4/GOT2 measured in mitochondrial-enriched lysates from infected myotubes. **(D)** Mitochondrial oxygen consumption (*J* O _2_) supported by pyruvate, malate, and ADP. **(E)** Rates myotube protein degradation (tyrosine release) with L-Kyn treatment was prevented Ad-PGC1α. **(F)** Quantification of myotube area (sarcomeric myosin). *p < 0.05, **p < 0.01, ***p < 0.001 using Student’s *t*-test in panels A–C. *p < 0.05, **p < 0.01, ****p < 0.0001 using two-way ANOVA with Tukey’s posthoc testing. N = 3-7 biologically independent C2C12 lines. Error bars in panels **(A–E)** represent SD. Panel F shows floating bars representing the minimum and maximum values with a line at the mean.

Previous studies have shown that exercise and expression of PGC1α in muscle can modulate KAT expression levels leading to enhanced biotransformation of L-Kyn to KA (Agudelo et al., 2014; Agudelo et al., 2019). Based on these observations, we sought to examine the impact of adenoviral-mediated expression of PGC1α on cultured myotubes treated with L-Kyn. Compared to Ad-GFP treated myotubes, Ad-PGC1α myotubes displayed significant increases in the mRNA levels of PGC1α (Figure 4A) as well as KAT1, KAT3, and KAT4/GOT2 (Figure 4B). Functionally, KAT4/GOT2 enzyme activity was also significantly increased (∼65%) in myotubes treated with Ad-PGC1α (Figure 4C). Consistent with results from L-Kyn fed mice, there was a significant decrease in mitochondrial respiratory capacity in Ad-GFP infected myotubes treated with 15 μM L-Kyn (Figure 4D). Ad-PGC1α treated myotubes not only displayed an increase in mitochondrial respiratory capacity compared to Ad-GFP myotubes in vehicle conditions (*p* < 0.05 vs. Ad-GFP in vehicle conditions), but the enhanced ability for L-Kyn degradation was sufficient to protect against the decrease in mitochondrial respiration following L-Kyn treatment (*p* < 0.0001 vs. Ad-GFP with L-Kyn treatment) (Figure 4D). In contrast to results in L-Kyn fed mice, Ad-GFP infected myotubes treated with L-Kyn had elevated protein degradation and atrophy (evidenced by smaller myotube area) compared to vehicle treated myotubes (Figures 4E,F). Importantly, Ad-PGC1α was found to abrogate these effects resulting in normal myotube size (Figures 4E,F).

## Discussion

Kynurenines have long been known to have neurobiological effects and elevated kynurenines have been linked with numerous neurological conditions (Hilmas et al., 2001; Schwarcz et al., 2012; Agudelo et al., 2014; Breda et al., 2016; Platten et al., 2019). Recent evidence has emerged linking L-Kyn accumulation with physical frailty, muscle atrophy and weakness, bone loss, and physical function (El Refaey et al., 2017; Kaiser et al., 2019a; Jang et al., 2020; Westbrook et al., 2020). Despite these striking associations, a direct causal relationship between L-Kyn accumulation and physical function/muscle health has not been fully established. In the current study, a diet model was used to elevate plasma L-Kyn and the L-Kyn/Trp ratio in young male mice. Importantly, the increase in the L-Kyn/Trp ratio in this mouse study (∼80%) is similar to the increase reported in aging and/or frail humans (∼40% increase in frail humans from Jang et al. (2020) and ∼63% increase in older adults from Westbrook et al. (2020)). This finding demonstrates the utility of a L-Kyn supplemented diet for preclinical work aiming to study pathologies related to L-Kyn accumulation.

One previous study in mice reported that young female mice that received daily injection of L-Kyn have lower muscle weights and myofiber size compared to controls injected with PBS (Kaiser et al., 2019a). However, L-Kyn injections in aged mice (22–24 mo old) did not suffer from atrophy. The latter finding may stem from the fact that, similar to aging humans, aged mice have elevated L-Kyn levels compared to younger mice (Westbrook et al., 2020; Wu et al., 2021). Consistent with this notion, treatment of aged mice with 1-methyl-d-tryptophan, an inhibitor of IDO which lowers L-Kyn levels, improved muscle size in this study. In contrast to their results, the present study did not find any changes in muscle weights, myofiber size, or contractile function. The underlying reasons for this discrepancy are unclear, but one potential contributing factor is the difference in the sex of rodents used between the studies (Rosa-Caldwell et al., 2021). Further, the study by Kaiser *et al.* did not report serum or plasma L-Kyn levels so it is not possible to compare the levels of L-Kyn between these studies. Nonetheless, atrophy and elevated proteolysis were detected in cultured myotubes treated with L-Kyn in the current study (Figure 4).

An interesting finding from the current study was that mice with elevated L-Kyn levels displayed an impairment in mitochondrial OXPHOS in skeletal muscle, which as mentioned above, occurred in the absence of muscle atrophy. It is possible that a longer dietary L-Kyn intervention could result in atrophy or that mitochondrial abnormalities precede atrophy in muscle. The latter statement is supported by a recent study showing that mitochondrial degeneration occurs before muscle atrophy in male, but not female, mice subjected to disuse atrophy (Rosa-Caldwell et al., 2021). Although the mechanisms underlying mitochondrial OXPHOS deficits driven by L-Kyn are unknown, previous studies have documented that L-Kyn has direct inhibitory effects on some mitochondrial enzymes (Baran et al., 2003; Thome et al., 2019). However, it is noteworthy that mitochondria are also a key source for the conversion of L-Kyn to KA and thus L-Kyn-mediated impairment in mitochondrial function could exacerbate the accumulation of L-Kyn, although a direct causal relationship has not been established yet.

Increasing the muscle’s capacity for L-Kyn degradation, either through genetic manipulation or physical exercise, has been shown to improve neurological outcomes (Agudelo et al., 2014). Further to this, transgenic expression PGC1α in muscle was also reported to enhance the utilization of L-Kyn resulting in improved malate aspartate shuttle function to enhance muscle energy transduction (Agudelo et al., 2019). Consistent with this notion, physical exercise/muscle contraction has been shown to alter kynurenine metabolism and the expression of KATs (Schlittler et al., 2016; Allison et al., 2019; Wyckelsma et al., 2020). Whereas KAT1 and KAT3 are found in the cytosol of muscle, KAT4/GOT2 is localized to the mitochondrial matrix. Similar to previous studies, we found that overexpression of PGC1α could enhance the levels of KAT transcripts and KAT4/GOT2 enzyme activity (Figure 4). In conjunction with these results, high functioning masters athletes that participate in vigorous physical activity routinely have preserved mitochondrial health compared to non-athlete controls, a feature that presumably leads to enhanced L-Kyn degradation (Ubaida-Mohien et al., 2022). This enhancement in L-Kyn degradation may limit the accumulation of L-Kyn and protect against the decline in muscle function.

A possible mechanism underlying the preservation of muscle function in conditions with enhanced muscle L-Kyn degradation may involve the motor neuron. The loss of motor neuron innervation is a key event that precipitates myofiber atrophy (Rowan et al., 2012; Hepple and Rice, 2016; Sonjak et al., 2019a). In *Drosophila*, mutations in the kynurenine pathway were reported to alter motor neuron function in flight muscles (Smirnov, 1997). Using motor neuron culture, Westbrook *et al.* reported that 3-hydroxykynurenine and quinolinic acid (downstream metabolites in the kynurenine pathway) depleted ATP levels, a feature that could contribute to the loss of motor neuron innervation *in vivo*. Taken together, these observations suggest that enhancing muscle L-Kyn degradation may preserve motor neuron health and function and thereby sustain muscle contractile performance. However, muscle function measured via motor neuron stimulation did not reveal functional impairment in the current intervention.

In summary, the current study establishes a L-Kyn supplemented diet model in mice that elevates L-Kyn and L-Kyn/Trp ratios to levels consistent with aging and frail humans. This 10-week intervention did not impact food intake, gross body weight, or muscle size. Impairments in muscle mitochondrial OXPHOS were detected suggesting that metabolic derangements precede functional and size impairments in muscle with elevated kynurenines.

## Data Availability

The original contributions presented in the study are included in the article/supplementary material, further inquiries can be directed to the corresponding author.
